# Flomoxef for neonates: extending options for treatment of neonatal sepsis caused by ESBL-producing Enterobacterales

**DOI:** 10.1093/jac/dkab468

**Published:** 2021-12-30

**Authors:** Christopher A Darlow, William Hope

**Affiliations:** Antimicrobial Pharmacodynamics and Therapeutics, University of Liverpool, Liverpool, UK; Antimicrobial Pharmacodynamics and Therapeutics, University of Liverpool, Liverpool, UK

## Abstract

**Background:**

Neonatal sepsis is a serious and frequently lethal infection, often complicated by antimicrobial resistance (including ESBLs) in low- and middle-income countries (LMICs). Flomoxef is an off-patent oxacephem β-lactam with stability against non-AmpC ESBLs, with potential for utility in these settings. To date, there has been no published flomoxef neonatal population pharmacokinetic (PopPK) model.

**Objectives:**

To summarize the clinical data available for flomoxef, build a neonatal PopPK model and assess the adequacy of different neonatal flomoxef regimens.

**Methods:**

A systematic literature search returned all available clinical or pharmacokinetic data of flomoxef use in neonates. Pharmacokinetic data were used to construct a PopPK model, with progressive incorporation of covariates into the final model. Monte Carlo simulations were performed using this final model to simulate drug exposures of different flomoxef regimens to calculate PTAs.

**Results:**

Individual-level clinical and pharmacokinetic data were extracted for 313 and 146 neonates, respectively, with population clinical data extracted for a further 199 neonates. Clinical and microbiological success rates were 89.71% and 82.8%, respectively, with minimal side effects. The final PopPK model incorporated body weight and postnatal age as covariates. PTA analyses predicted that IV regimens of 20 mg/kg q12h, 20 mg/kg q6–8h and 40 mg/kg q6–8h are adequate for neonates aged 0–7, 7–14 and 14–28 days, respectively.

**Conclusions:**

To the best of our knowledge, this is the first published neonatal PopPK model for flomoxef. Given the high treatment success rates, low toxicity rates and off-patent status, this drug has potential for use in the treatment of neonatal sepsis in ESBL-prevalent LMIC settings.

## Introduction

Flomoxef is a β-lactam antibiotic first synthesized in Japan in the 1980s.^1–3^ Like other β-lactam antibiotics, it acts by binding to one or more unspecified PBPs, interfering with bacterial cell wall synthesis. Specifically, flomoxef is a synthetic oxacephem (oxacephems being derivatives of the cephalosporin class of antibiotics) that contains side chains that confer activity against Gram-positive and Gram-negative bacteria as well as anaerobes. These side chains also provide stability to hydrolysis by some β-lactamases,^3^ which remain the most prevalent resistance mechanism against β-lactam antibiotics. While the oxacephems typically have several idiosyncratic toxicities, which include a coumarin-like coagulopathy and disulfiram-like effects, flomoxef is devoid of these adverse effects.^3^

Neonatal sepsis is a common serious and frequently lethal infection of infants that is caused by a wide range of pathogens, including *Streptococcus agalactiae* (or group B streptococci), Gram-negative organisms (e.g. *Escherichia coli* and *Klebsiella pneumoniae*) and *Staphylococcus aureus*.^4^ Globally, it causes an estimated 430 000–680 000 deaths annually, predominantly in low- and middle-income countries (LMICs).^5^^,^^6^ Currently, the WHO recommends empirical treatment of neonatal sepsis with a combination of a narrow-spectrum β-lactam and gentamicin.^7^^,^^8^ However, this regimen is increasingly compromised by rising levels of antimicrobial resistance (AMR)—in particular, high levels of ESBLs and aminoglycoside modifying enzymes (AMEs).^9–11^ In this context, alternative agents for the empirical treatment of neonatal sepsis are urgently required.

Flomoxef has several properties that make it a potentially suitable agent for the treatment of neonatal sepsis in LMICs where ESBLs are prevalent. Flomoxef is stable to degradation by ESBLs (except for Ambler class C enzymes, e.g. AmpC),^12^ it is potentially affordable in LMIC settings and it has a favourable safety profile.^2^ Importantly, however, flomoxef is only licensed in Japan, South Korea, China and Taiwan. A neonatal population pharmacokinetic (PopPK) model of flomoxef has not been published. We retrieved the available published clinical and pharmacokinetic data from neonates, summarized the clinical data and fitted a PopPK model to the pharmacokinetic data. Monte Carlo simulations and target attainment analyses were performed to assess the adequacy of different candidate regimens for the treatment of neonatal sepsis.

## Methods

### Data extraction

Publications were identified by searching Medline using the terms ‘flomoxef’ or ‘6315-S’ (the development name for flomoxef at Shionogi). Abstracts for all papers were screened by the authors for relevance to neonates as defined by use of flomoxef in infants aged <6 months with clinical and/or pharmacokinetic data collected. The following covariates were extracted (where available): dosage, length of treatment, postnatal age, sex, body weight at time of administration, birth weight, gestation, diagnosis, pathogen, comorbidities, clinical success, microbiological success, adverse reactions, pharmacokinetic time profiles and other pharmacokinetic parameters. Where papers were not in English, translation software (Google Translate) was used to extract the relevant data.

### Data analysis

Frequentist and descriptive statistical analysis of demographic and clinical outcome data was performed using Microsoft Excel and SPSS (IBM Corp., Armonk, NY, USA; v27). For comparisons of means, either two-sample t-tests or Welch’s t-tests were used, dependent on equivalence of variance.

### Population pharmacokinetic modelling

Pharmacokinetic data from individuals with individual time–concentration profiles, details of administered flomoxef regimens and available current body weight and age covariates were modelled using a non-parametric population methodology with Pmetrics.^13^ The following two-compartment structural base model was used:

$$\frac{dX(1)}{dt}=\mathrm{RateIV}\left(1\right)-\left(\left(\frac{Cl}{V}+\mathrm{KCP}\right)\times \;X\left(1\right)\right)+(\mathrm{KPC}\;\times \;X\left(2\right))$$

 

$$\frac{dX(2)}{dt}=(\mathrm{KCP}\times X\left(1\right))-(\mathrm{KPC}\;\times \;X\left(2\right))$$

X(1) and X(2) are the amounts of flomoxef in the central and peripheral compartments, respectively, RateIV(1) is the rate of administration of flomoxef into the central compartment, Cl is the first-order clearance of flomoxef from the central compartment, V is the volume of the central compartment and KCP and KPC are first-order intercompartmental rate constants for drug transfer between the central and peripheral compartments.

The goodness-of-fit of the model was assessed using a combination of: (i) r^2^ coefficient of determination from the linear regression in the observed–predicted plots for both population and individual parameter estimates; (ii) minimization of bias and imprecision in the observed–predicted plots for both population and individual parameter estimates; (iii) log-likelihood values; (iv) Akaike Information Criterion (AIC); and (v) Bayesian Information Criterion (BIC).

Potential associations between covariates and Bayesian estimates of Cl and V from each patient were explored visually and assessed statistically using stepwise linear regression. Covariates were progressively incorporated into the base model, with successful incorporation defined by improvement of the model performance, as assessed by improvement of the goodness-of-fit parameters and statistical significance of difference from the previous model (multivariate two-sample test based on k-nearest neighbours) and removal of the statistical association between covariates and pharmacokinetic parameters.

First, body weight (standardized to a 70 kg adult) was allowed to affect both Cl and V (Eqn 1). A ‘Std’ subscript denotes the weight-normalized estimate for each value and assumes the value estimated for a 70 kg adult. A standard allometric scaling exponent of 0.75 was included for clearance into the model to describe the non-linear relationship between weight (as a proxy for size) and clearance.^14^
 

$$\left(1\right)\;Cl={Cl}_{\mathrm{Std}}\;\times \;{\left(\frac{\mathrm{Weight}}{70}\right)}^{0.75}$$

 

$$V=V_{\mathrm{Std}}\;\times \;(\frac{\mathrm{Weight}}{70})$$

With this approach the parameter V_Std_ became independent from all covariates and the goodness-of-fit of the model improved. However, there were residual relationships between Cl_Std_ and both weight (kg) and age (days). The latter was further added into the clearance parameter definition, with the term ‘Age + 1’ (to avoid zero values) with scaling exponent ‘A’ estimated directly from the data (Eqn 2):

$$\left(2\right)\;Cl={Cl}_{\mathrm{Std}}\;\times \;{\left(\frac{\mathrm{Weight}}{70}\right)}^{0.75}\;\times \;{\left(\mathrm{Age}+1\right)}^{A}$$

 

$$V=V_{\mathrm{Std}}\;\times \;(\frac{\mathrm{Weight}}{70})$$

This further improved the predictive performance of the model, but there was still a residual relationship between Cl_std_ and weight. Therefore, an additional exponent scaler—‘B’—was added to the body weight parameter (Eqn 3). Body weight is co-correlated with gestation and, in the absence of complete gestation data, this additional scaler likely accounts for the ontogeny of renal function not captured by postnatal age alone (i.e. that of gestational age).

$$\left(3\right)\;Cl={Cl}_{\mathrm{Std}}\;\times \;{\left(\frac{\mathrm{Weight}}{70}\right)}^{\left(0.75+B\right)}\;\times \;{\left(\mathrm{Age}+1\right)}^{A}$$

 

$$V=V_{\mathrm{Std}}\;\times \;(\frac{\mathrm{Weight}}{70})$$

This final structural model further improved model performance and represents the final model.

### Monte Carlo simulations

Using the final structural model, a Monte Carlo simulation of 10 000 neonates was performed using Pmetrics.^13^ Different flomoxef regimens and age brackets (0–7, 7–14 and 14–28 days) were simulated. Body weight distribution was simulated using a correlation matrix between covariates and Bayesian posterior parameter values, informed by the final model.

The PTA of each simulated baby was calculated. The pharmacodynamic targets were derived from a recent mouse-thigh model study of flomoxef.^15^ These targets were 40% free drug concentration time above MIC (*fT*_>MIC_) for 1 log bacterial kill and 25% *fT*_>MIC_ for bacterial stasis. The PTA was determined for each flomoxef regimen in each age group.

## Results

### Literature review

Twelve publications were identified with flomoxef administered to neonates with clinical outcome and/or pharmacokinetic outcome data (Table S1, available as Supplementary data at *JAC* Online).^16–27^ Individual-level data were available for 313 and 146 neonates for clinical outcome and pharmacokinetic data, respectively. None of these 313 and 146 neonates overlapped (i.e. no individual provided data for both pharmacokinetic and clinical outcome data). Further population-level (i.e. pooled data) clinical outcome data were retrieved for an additional 199 neonates. All pharmacokinetic papers were of similarly high quality, as assessed by the ClinPK reporting standards,^28^ with similar methodologies, and therefore deemed suitable for collation. All clinical outcome data were from non-controlled and non-randomized studies with primary aims to validate the efficacy and safety of flomoxef demonstrated in older age groups in neonates.

### Clinical outcomes

The demographics of the individuals for whom clinical outcomes are available are shown in Table S2. Comparable demographic data are not available for the single study that contributed the population-level clinical outcome data.^19^ The combined outcome data of both individual- and population-level data are shown in Table 1. A wide variety of clinical indications were described. Excluding the use of flomoxef for prophylaxis, the most common clinical indications were pneumonia, chorioamnionitis, neonatal sepsis and skin and soft tissue infection (SSTI). Pathogens were not identified in 50.78% of cases. When described, *S. aureus* and *E. coli* were the most commonly encountered pathogens.

**Table 1.** Clinical neonatal outcome data grouped by individual-level data, population-level data and total

Characteristic	Individual-level data (*n*)	Population-level data (*n*)	Total (%)
Infection indication for treatment			
bronchitis	10	7	17 (3.32%)
chorioamnionitis	46	46	92 (17.97%)
CNS infection	4	2	6 (1.17%)
ENT infection	4	–	4 (0.78%)
meconium aspiration	3	–	3 (0.59%)
neonatal sepsis	47	37	84 (16.41%)
pneumonia	59	60	119 (23.24%)
prophylaxis	92	–	92 (17.97%)
SSTI	24	27	51(9.96%)
urinary tract infection	15	15	30 (5.86%)
viral	3	–	3 (0.59%)
abdominal infection	2	–	2 (0.39%)
other	4	5	9 (1.76%)
Identified pathogen			
*S. aureus*	32	29	61 (11.91%)
*S. agalactiae*	5	4	9 (1.76%)
other *Staphylococcus* spp.	7	4	11 (2.15%)
*E. coli*	23	20	43 (8.40%)
*Streptococcus pneumoniae*	2	1	3 (0.59%)
*Haemophilus influenza*	5	2	7 (1.37%)
*Enterococcus faecalis*	3	3	6 (1.17%)
other Gram-negative	6	10	16 (3.13%)
mixed	1	–	1 (0.20%)
not identified	134	126	260 (50.78%)
no suspected bacterial infection	95	–	95 (18.41%)
Clinical success			
yes	184	191	375 (89.71%)
no	13	8	21 (5.02%)
unknown	22	–	22 (5.26%)
Microbiological success			
yes	67	63	130 (82.80%)
no	5	3	8 (5.10%)
unknown	12	7	19 (12.10%)
Side effects			
diarrhoea	5	4	9 (1.76%)
eosinophilia	7	13	19 (3.91%)
thrombocytosis	5	3	8 (1.56%)
raised liver enzymes	11	16	27 (5.27%)
anaemia	–	1	1 (0.20%)

Percentages for clinical success include only bacterial infective indications and percentages for microbiological success include only individuals where a bacterial pathogen was identified. Side-effect percentages are of the total population. All population-level data were sourced from Fujii *et al*.;^19^ all individual-level data were sourced from the other 10 included studies.^16^^,^^17^^,^^20–27^

Clinical and microbiological success was relatively high with overall success rates of 89.71% and 82.80%, respectively, for indications involving a confirmed or suspected bacterial infection. Of the prevalent indications, clinical failures occurred in 9/119 (7.6%) pneumonia cases, 3/84 (3.6%) neonatal sepsis cases and 5/51 (9.8%) SSTI cases. A further 2/119 (1.7%) pneumonia cases and 2/84 (2.4%) sepsis cases had unknown clinical outcomes (all SSTI cases were accounted for). The clinical failures and unknown outcomes were from different source datasets, none of which gave defined case definitions.

Of the prevalent pathogens, microbiological failures occurred in 2/61 (3.3%) infections caused by *E. coli* and 2/43 (4.7%) infections caused by *S. aureus*. The regimens used in the collated data were highly variable, reflecting the uncertainty of the neonatal regimen in the early 1990s. Schedules of administration were almost always q6–12h, but dosages varied from 10 to 93 mg/kg. The occurrence of side effects appeared to be idiosyncratic with no significant differences in the daily dose received by neonates with and without adverse events (*P* = 0.33, Welch’s t-test). No recorded adverse effects were classified as severe.

### Pharmacokinetic data

All available data from the 146 babies with reported individual-level pharmacokinetic data were extracted. Data regarding dose, age, β-half-life (calculated by the study authors) and serial flomoxef serum concentrations were extracted for all individuals. All flomoxef concentrations in all studies were determined by microbiological bioanalytical methods described by Kimura *et al*.,^29^ with a limit of quantification of 0.2 mg/L. No individual datapoint was below this value.

Incomplete data were available for body weight (106/146), birth weight (82/146), gestation (77/146) and sex (102/146) covariates. For the 31/40 patients aged ≤3 days and without body weight data, birth weight was assumed as their current weight for the purposes of modelling. Full demographic data for this pharmacokinetic cohort are provided in Table S3. Patients with available age and body weight covariates were selected for modelling rather than interpolation of missing values for the entire population.

### Population pharmacokinetic modelling

A base structural model was fitted to the pharmacokinetic data obtained from the 137 babies with full age and body weight data. This base structural model demonstrated statistically significant associations between body weight and Cl and V parameters (*P* < 0.05). Incorporation of body weight into the definition of Cl and V (Eqn 1 in the Methods section) removed any association of covariates with V. However, the postnatal age and body weight covariates became/remained associated with Cl_Std_ (*P* < 0.05). Incorporation of postnatal age into the Cl parameter definition (Eqn 2) removed any significant association between age and Cl_Std_; however, statistical association remained between body weight and Cl_Std_ (*P* < 0.05). Incorporation of an additional scalar of body weight in the definition of Cl (Eqn 3) removed all statistically significant association between covariates and Cl_Std_ and V_Std_ parameters.

**Figure 1.**
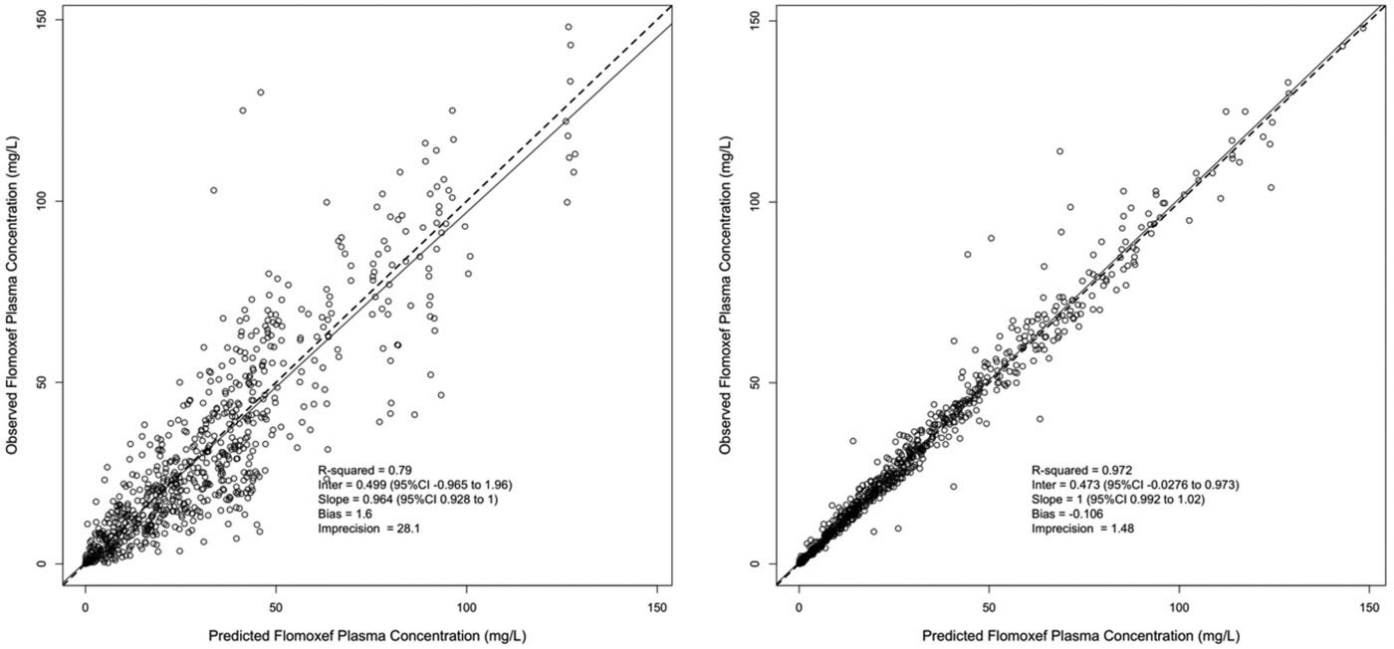
Observed versus predicted plots for the final model for population (left) and individual (right) Bayesian posteriors. The broken line indicates the observed versus predicted regression line of the model and the continuous line indicates a hypothetical perfect 1:1 observed versus predicted regression line.

This final model had a high r^2^ value (0.972 and 0.79 for individual and population models, respectively) and acceptably low bias and imprecision (Figure 1). These values and log-likelihood values, AICs and BICs were all improved from base structural and earlier models incorporating covariates (Table S4). The final model was also validated by a prediction-corrected visual predictive check (pcVPC) (Figure S1). Median and mean parameter values with 95% credibility intervals of this final model are shown in Table 2.

**Table 2.** Median, mean and variance parameter values from the final PopPK model

Parameter	Median (95% credibility interval)	Mean	SD
Cl_Std_ (L/h/70 kg)	24.059 (21.667–30.842)	23.759	10.483
V_Std_ (L/70 kg)	19.595 (18.23–21.585)	22.499	13.361
KCP (h^−1^)	2.770 (1.709–4.696)	5.890	6.516
KPC (h^−1^)	7.007 (2.438–13.201)	11.443	10.764
A	0.350 (0.299–0.458)	0.400	0.233
B	0.744 (0.695–0.790)	0.693	0.260

Cl_Std_ and V_Std_ are estimated standardized clearances and volume of the central compartment values, adjusted for weight ± age. KCP and KPC are kinetic constants determining movement between the central and peripheral compartments and vice versa. A and B are the estimated exponents for weight and age for clearance.

### PTA

**Figure 2.**
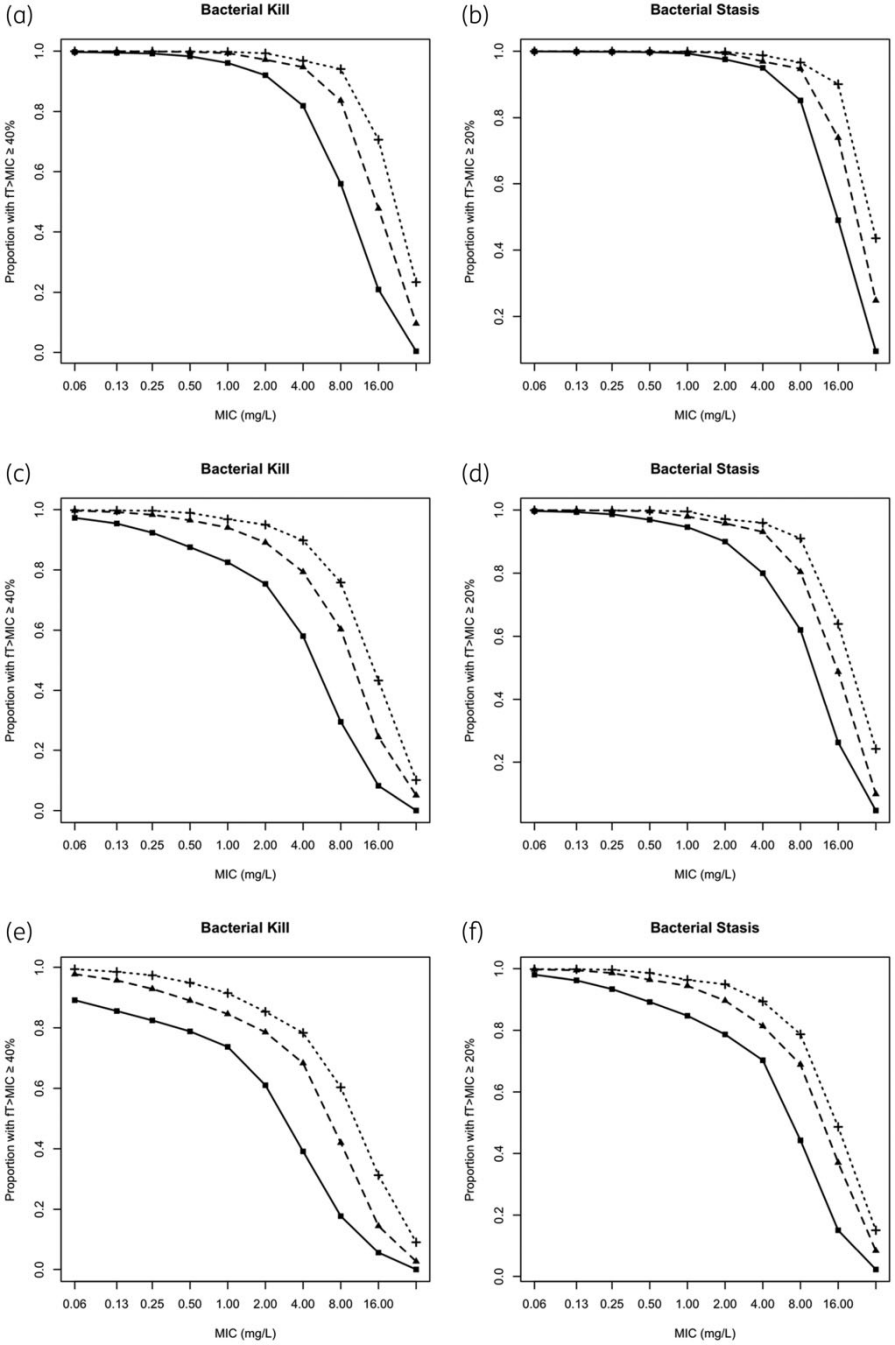
PTA of q6h, q8h and q12h schedules of 20 mg/kg flomoxef in neonates aged 0–7 days (a and b), 7–14 days (c and d) and 14–28 days (e and f) for bactericidal kill (a, c and e) and stasis (b, d and f) for an MIC range of 0.0625–32 mg/L. Squares, 20 mg/kg q12h; triangles, 20 mg/kg q8h; crosses, 20 mg/kg q6h.

PTA analyses were performed with different dosages (10–40 mg/kg) and schedules (q6–12h) in different age groups. For neonates aged 0–7 days, a 20 mg/kg q12h regimen achieved >95% target attainment at an MIC of 1 mg/L for bacterial kill and 2 mg/L for bacterial stasis (Figure 2a and b). For neonates aged 7–14 days, a 20 mg/kg q12h regimen achieved >95% target attainment at MIC values of 0.125 and 0.5 mg/L for kill and stasis, respectively. Increasing the schedule to q8h increases the MIC at which >95% target attainment is achieved to 0.5 and 2 mg/L for kill and stasis, respectively. Increasing the schedule further to q6h increases these MIC thresholds for >95% target attainment to 2 and 4 mg/L, respectively (Figure 2c and d). For neonates aged 14–28 days, a dose of 20 mg/kg required a schedule of q6h to achieve >95% target attainment at MIC values of 0.25 and 1 mg/L for kill and stasis, respectively (Figure 2e and f).

In all age groups and schedules, increasing the dose to 40 mg/kg improves the MIC at which >95% PTA occurs by 2-fold, e.g. to 2 and 4 mg/L for kill and stasis, respectively, in neonates aged 0–7 days with a q12h schedule.

## Discussion

To the best of our knowledge, this is the first published neonatal PopPK model of flomoxef. Although underused in a global context, with use largely restricted to East Asia, its stability to non-AmpC ESBLs, off-patent status and favourable toxicity profile make it an attractive option for use more widely and in the context of neonatal sepsis caused by increasingly resistant pathogens. The collated clinical and microbiological treatment success rate in neonates was high across a range of conditions, even with highly variable regimens.

The recommended regimens listed in the Summary of Product Characteristics (SPC) for flomoxef are variable,^30^ recommending 20 mg/kg q8–12h up to 3 days of life, with a q6–8h schedule at older ages. However, a less frequent schedule renders the drug more useful in LMIC settings where resources to facilitate IV drug administration are limited. There are multiple published epidemiological studies with flomoxef MIC data, with estimates for a flomoxef MIC_90_ of 0.5–1 mg/L and MIC_50_ (when estimated) of 0.125 mg/L for Enterobacterales without plasmid-mediated AmpC ESBL genes.^31–36^ Although an epidemiological cut-off value cannot be formally calculated with the MIC data that are available, it is unlikely to be >1 mg/L.

A recent study has defined the pharmacodynamic target for flomoxef to be 40% *fT*_>MIC_ for 1 log bacterial kill and 25% *fT*_>MIC_ for bacterial stasis.^15^ Our flomoxef PopPK model demonstrates that these targets can be achieved for an MIC of 1 mg/L with a 20 mg/kg q12h regimen for neonates aged 0–7 days and a 20 mg/kg q6–8h regimen for neonates aged 7–14 days. For neonates aged 14–28 days, a 20 mg/kg q6h or 40 mg/kg q6–8h regimen is required, depending on the precise epidemiological cut-off value and tolerance for only achieving the stasis pharmacodynamic target. On current evidence, we suggest that these regimens are suitable for further study in the respective neonatal age groups.

There are several limitations to these analyses. In the absence of complete covariate data, we relied on body weight and age covariates alone to account for the variability of the data that may be attributable to unavailable covariates (e.g. gestational age and renal function). For example, the addition of a second exponent (B) is likely a reflection of the ontogeny associated with prematurity, which is co-correlated with body weight, but not postnatal age. While a total exponent of body weight >1 accounts for these neonatal data well, it is clearly not compatible with the likely pharmacokinetics for older/heavier individuals. Therefore, the predictive value of this model only applies to neonates and cannot be extrapolated beyond this population. Consequently, the PTA analysis is only relevant to neonates with a postnatal age of ≤28 days. We did consider interpolating missing gestational age covariate values to prevent this issue, but the high levels of missing data (47.3%) meant that this was inappropriate. Given the age of the studies (all are >28 years old), contacting the study authors to provide the missing data was not feasible.

Secondly, the individuals included are entirely of Japanese ethnicity. While flomoxef is predominantly renally excreted,^2^ 10%–15% of the drug is metabolized,^37^ predominantly to the inactive metabolite hydroxyethyl-tetrazolethinol. The specific enzymes and any potential transporters of flomoxef have not been identified. Therefore, there may be differences in metabolism related to ethnicity that require further study. Additionally, the association between weight and ontogeny may be different in non-Japanese populations. Therefore, some caution must be used in extrapolating to non-Japanese ethnic groups, in the absence of further pharmacokinetic data obtained from other ethnic groups.

Despite potential limitations, flomoxef is a potentially useful agent to treat neonatal sepsis in LMIC settings with increasingly prevalent AMR (particularly mediated by ESBLs produced by Enterobacterales pathogens) and this study and our analyses suggest the neonatal regimens for different age groups that adequately achieve the recently defined pharmacodynamic targets.

## Supplementary Material

dkab468_Supplementary_Data
