# Correction to: Flomoxef for neonates: extending options for treatment of neonatal sepsis caused by ESBL-producing Enterobacterales

**DOI:** 10.1093/jac/dkac153

**Published:** 2022-05-31

**Authors:** Christopher A Darlow, William Hope

*Journal of Antimicrobial Chemotherapy*, Volume 77, Issue 3, March 2022, Pages 711–718, https://doi.org/10.1093/jac/dkab468

Our original paper^1^ reported values for the model parameters A and B in Table 2 incorrectly, with the values inadvertently switched. The corrected table is shown here. All models and simulations in the original paper^1^ and subsequent correspondence^2^ used the correct values and the conclusions of the paper remain unaltered. The correspondence received relating to the original paper^3^ used the original incorrectly switched values for parameters A and B, and should be interpreted with this in mind. The online version of the article has now been corrected.

**Table 2.** Median, mean and variance parameter values from the final PopPK model

Parameter	Median (95% credibility interval)	Mean	SD
Cl_Std_ (L/h/70 kg)	24.059 (21.667–30.842)	23.759	10.483
V_Std_ (L/70 kg)	19.595 (18.23–21.585)	22.499	13.361
KCP (h^−1^)	2.770 (1.709–4.696)	5.890	6.516
KPC (h^−1^)	7.007 (2.438–13.201)	11.443	10.764
A	0.350 (0.299–0.458)	0.400	0.233
B	0.744 (0.695–0.790)	0.693	0.260

Cl_Std_ and V_Std_ are estimated standardized clearances and volume of the central compartment values, adjusted for weight±age. KCP and KPC are kinetic constants determining movement between the central and peripheral compartments and vice versa. A and B are the estimated exponents for age and weight for clearance.

The authors apologise for this error.
