# Supplementary material for: Flomoxef for neonates: extending options for treatment of neonatal sepsis caused by ESBL-producing Enterobacterales
Source: J Antimicrob Chemother. 2021 Dec 30;77(3):711–8. doi: 10.1093/jac/dkab468 (PMC8864998; doi:10.1093/jac/dkab468)
Supplement: dkab468_Supplementary_Data [file dkab468_supplementary_data.docx]

**Supplementary data**

| **Publication** | **Individuals with clinical outcome data (n)** | | **Individuals with PK data (n)** | **Location** |
| --- | --- | --- | --- | --- |
|  | **Individual level data** | **Population level data only** |  |  |
| **Okura et al** ^1^ | 5 | - | 10 | Kobe, Japan |
| **Tabuki et al** ^2^ | 6 | - | - | Osaka, Japan |
| **Fujita et al** ^3^ | 43 | - | 19 | Asahikawa, Japan |
| **Iwai et al** ^4^ | 78 | - | 27 | Meitetsu , Japan |
| **Motohiro et al** ^5^ | 98 | - | 15 | Kurume, Japan |
| **Ihara et al** ^6^ | 21 | - | 4 | Mie, Japan |
| **Tomimasu et al** ^7^ | 21 | - | 10 | Nagasaki, Japan |
| **Akita et al** ^8^ | 21 | - | 5 | Yamato, Japan |
| **Azagami et al** ^9^ | 16 | - | 40 | Keio, Japan |
| **Sato et al** ^10^ | 4 | - | 8 | Tokyo, Japan |
| **Kimura et al** ^11^ | - | - | 8 | Juntendo, Japan |
| **Fujii et al** ^12^ | - | 199 | - | Multiple locations, Japan |
| **Total** | **313** | **199** | **146** |  |

***Table S1.*** *Summary of published clinical outcome and pharmacokinetic data for flomoxef in neonates.*

| **Characteristic** | **Median value (SD)** | **Range** | **N** |
| --- | --- | --- | --- |
| M:F split | 54.3:45.7 | N/A | 313 |
| Age (Days) | 2 (17.81) | 0 – 138 | 313 |
| Weight (g) | 2920 (935.59) | 797 – 9000 | 292 |
| Birth Weight (g) | 2926 (716.25) | 780 – 4180 | 145 |
| Gestation (weeks) | 39 (3.18) | 25 – 42 | 126 |
| Dose received (mg/kg) | 23 (9.80) | 10 – 93 | 313 |
| Dosing schedule:  q24h  q12h  q8h  q6h | 1  85  153  74 | N/A | 313 |

***Table S2.*** *Demographics for 313 neonatal patients receiving flomoxef therapy with clinical data available. Data for some variables available for only a proportion of the total.*

| ***Characteristic*** | ***Median (SD)*** | ***Range*** | ***N*** |
| --- | --- | --- | --- |
| *Dose (mg/kg)* | *20 (9.61)* | *9.82 - 40* | *146* |
| *Age (days)* | *7 (19.30)* | *0 - 138* | *146* |
| *Body Weight (g)* | *2900 (745.59)* | *1112 - 5000* | *137* |
| *Birth Weight (g)* | *2702.5 (702.45)* | *800 - 4100* | *82* |
| *Gestation (weeks)* | *38 (3.51)* | *25 – 43* | *77* |
| *β-phase T_1/2_ (Hours)* | *2.22 (1.28)* | *0.68 – 6.6* | *146* |
| *M:F* | *60:42* |  | *102* |

***Table S3.*** *Demographic data of 146 neonatal patients with available PK data. Some data had incomplete availability; the final column details the numbers of individuals for which each variable was available.*

| ***Model*** | ***R^2^ value (post / pop)*** | ***Bias***  ***(post / pop)*** | ***Imprecision (post / pop)*** | ***-2*Log-likelihood*** | ***AIC*** | ***BIC*** |
| --- | --- | --- | --- | --- | --- | --- |
| *Base* | *0.77 / 0.519* | *0.11 / 3.52* | *14.2 / 100.94* | *4095* | *4109* | *4141* |
| *+ Eq. 1* | *0.981 / 0.713* | *-0.02 / 3.31* | *0.97 / 62.34* | *4138* | *4153* | *4185* |
| *+ Eq. 2* | *0.978 / 0.755* | *-0.09 / -0.15* | *1.32 / 24.24* | *4229* | *4239* | *4262* |
| *+ Eq. 3* | *0.972 / 0.79* | *-0.11 / 1.6* | *1.48 / 28.08* | *4258* | *4268* | *4291* |

***Table S4.*** *Model performance of base structural and sequential models with progressively incorporated covariates, as described in the model development methodology. Post = relating to individual Bayesian posterior model; pop = relating to population model; AIC = Akaike information criterion; BIC = Bayesian information criterion*

**
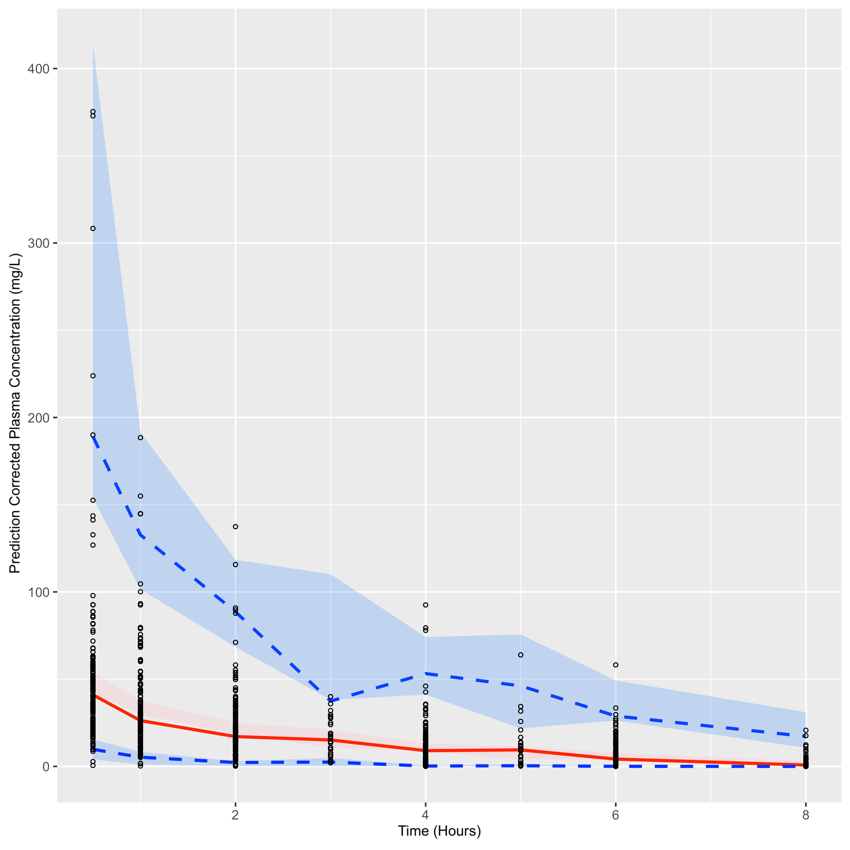
**

***Figure S1.*** *Prediction corrected Visual Predictive Check (pcVPC) of flomoxef neonatal model. Red solid line = observed data median value; Blue dash lines = 2.5% and 97.5% confidence interval of observed data; Pink shaded area = Simulated estimate of median value; Blue shaded area = simulated estimate of 2.5% and 97.5% confidence intervals; open circles = observed data points corrected for dose.*

**References**

1. Okura K, Yamakawa M, Kuroki S, Haruta T, Kobayashi Y. [Clinical evaluation of flomoxef in neonatal infections]. *Jpn J Antibiot* 1991; **44**: 1286–93. Available at: http://www.ncbi.nlm.nih.gov/pubmed/1784078.

2. Tabuki K, Nishimura T. [Clinical studies on flomoxef in neonates]. *Jpn J Antibiot* 1993; **46**: 539–46. Available at: http://www.ncbi.nlm.nih.gov/pubmed/8371490.

3. Fujita K, Murono K, Saijyo M, *et al.* [Flomoxef in neonates and young infants; clinical efficacy, pharmacokinetic evaluation and effect on the intestinal bacterial flora]. *Jpn J Antibiot* 1991; **44**: 1216–27. Available at: http://www.ncbi.nlm.nih.gov/pubmed/1784072.

4. Iwai N, Nakamura H, Miyazu M, *et al.* [Laboratory and clinical evaluations of flomoxef sodium in neonates]. *Jpn J Antibiot* 1991; **44**: 1265–85. Available at: http://www.ncbi.nlm.nih.gov/pubmed/1784077.

5. Motohiro T, Maruoka T, Nagai K, *et al.* [Laboratory and clinical studies on flomoxef in neonates and premature infants]. *Jpn J Antibiot* 1993; **46**: 547–67. Available at: http://www.ncbi.nlm.nih.gov/pubmed/8371491.

6. Ihara T, Kamiya H, Matsuda T, *et al.* [Pharmacokinetic and clinical evaluations of flomoxef in neonates]. *Jpn J Antibiot* 1991; **44**: 1259–64. Available at: http://www.ncbi.nlm.nih.gov/pubmed/1784076.

7. Tomimasu K, Tsuji Y, Fukuda M, *et al.* [Pharmacokinetic and clinical studies on flomoxef in mature and premature infant]. *Jpn J Antibiot* 1991; **44**: 1294–302. Available at: http://www.ncbi.nlm.nih.gov/pubmed/1784079.

8. Akita H, Sato Y, Iwata S, Sunakawa K, Yokota T. [Pharmacokinetic and clinical studies on flomoxef in neonates and premature infants]. *Jpn J Antibiot* 1991; **44**: 1240–9. Available at: http://www.ncbi.nlm.nih.gov/pubmed/1784074.

9. Azagami S, Isohata E, Takeda S, *et al.* [Pharmacokinetics and clinical efficacy of flomoxef in neonates]. *Jpn J Antibiot* 1991; **44**: 1228–39. Available at: http://www.ncbi.nlm.nih.gov/pubmed/1784073.

10. Sato H, Narita A, Matsumoto K, *et al.* [Studies of flomoxef in neonates]. *Jpn J Antibiot* 1991; **44**: 1250–8. Available at: http://www.ncbi.nlm.nih.gov/pubmed/1784075.

11. Kimura K, Miyano T, Shimomura H. [Pharmacokinetic studies of flomoxef in the neonatal field]. *Jpn J Antibiot* 1991; **44**: 1303–6. Available at: http://www.ncbi.nlm.nih.gov/pubmed/1784080.

12. Fujii R, Fujita K, Murono K, *et al.* [Pharmacokinetics and clinical studies on flomoxef in neonates and premature infants. A study of flomoxef in the perinatal collaboration research group]. *Jpn J Antibiot* 1993; **46**: 518–38. Available at: http://www.ncbi.nlm.nih.gov/pubmed/8371489.
